# Institutional Defalcations : The Assistant-Secretary's Responsibility

**Published:** 1914-06-13

**Authors:** 


### THE EDITOR'S LETTER-BOX.

### Institutional Defalcations: The Assistant-Secretary's Responsibility.

*To the Editor of* The Hospital.

Sir,—The following question is one on which I should be glad to have the opinion of yourself and your readers. It is one which I have heard asked several times of late. Supposing a head administrator, no matter whether designated medical superintendent, house governor, or simple secretary, be responsible for a defalcation in his hospital's accounts, how far must his assistant secretary be considered to share his responsibility? Ask most senior hospital managers whom one meets and they will tell you that it would have been impossible for themselves or anyone else to have tampered with the accounts without the fact having been discovered within twenty-four hours.

Luckily financial misdemeanours are rare in the hospital world, although I see this week that a certain provincial hospital treasurer is at present on trial in connection with a charge of converting funds to his own use; but it is clear that the younger men, the assistants who have their careers to make, are much concerned in the question. If it be generally held that they cannot fail to know whatever is being done with the finance at the officer's disposal it is clear that their own future will be compromised, as well as that of their chief, in the event of a defalcation being discovered. In other words, the door to promotion will be closed to them. A well-known administrator said to me the other day, "If an assistant secretary really knows his business the whole of the hospital's finance will be at his fingers' ends, and he cannot fail immediately to detect anything that may be wrong. If he does not have this complete knowledge he is clearly unfit for promotion."

These last are strong words, and might seem decisive; but there is also a feeling among certain boards that I could name that an assistant secretary should not be held responsible for any acts other than his own, and may be fit for promotion, provided, of course, he has committed no offence. Can your readers enlighten me as to the better and more responsible opinion?

Yours faithfully,
Committeeman.

[*₊* We invite correspondence upon the points raised by our contributor. Now the training of men for the higher posts in our hospitals is proceeding, it is time that the moralities were defined and enforced during training and afterwards. Discipline is sometimes too lax.—Ed. The Hospital.]

### A Question of Professional Conduct.

*To the Editor of* The Hospital.

